# Bis(3,5-dimeth­oxy­phen­yl)phosphinic acid

**DOI:** 10.1107/S1600536811008440

**Published:** 2011-03-15

**Authors:** Wei Cheng, Zhi-Qiang Feng, Jun-Mei Tang

**Affiliations:** a Department of Biological and Chemical Engineering, Chien-Shiung Institute of Technology, Taicang 215411, Suzhou, People’s Republic of China; b School of Material Engineering, Jinling Institute of Technology, Nanjing 211169, People’s Republic of China

## Abstract

In the crystal structure of the title compound, C_16_H_19_O_6_P, inter­molecular O—H⋯O inter­actions link the mol­ecules into chains parallel to the *b* axis. These chains are linked by C—H⋯π and π–π inter­actions [centroid–centroid distance = 3.7307 (29) Å] into a three-dimensional network. The dihedral angle between the benzene rings is 73.5 (1)°. The C and O atoms of all four methoxy groups lie very close to the mean planes of their attached rings; the C atoms are 0.055 (2)–0.1038 (1) Å out of the mean plane of the attached rings.

## Related literature

For standard bond lengths, see: Allen *et al.* (1987 ▶). For the synthesis of the title compound, see: Watson *et al.* (2006 ▶).
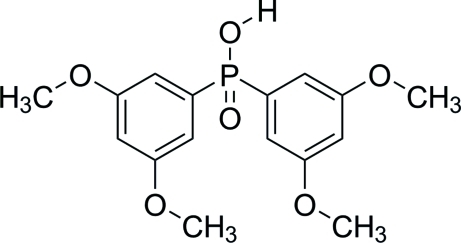


## Experimental

### 

#### Crystal data

C_16_H_19_O_6_P
*M* *_r_* = 338.28
Monoclinic, 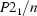

*a* = 14.554 (3) Å
*b* = 7.7620 (16) Å
*c* = 14.634 (3) Å
β = 96.14 (3)°
*V* = 1643.7 (6) Å^3^
*Z* = 4
Mo *K*α radiation
μ = 0.20 mm^−1^
*T* = 298 K
0.30 × 0.20 × 0.10 mm

#### Data collection

Enraf–Nonius CAD-4 diffractometer
Absorption correction: ψ scan (North *et al.*, 1968 ▶) *T*_min_ = 0.944, *T*_max_ = 0.981
3138 measured reflections
3014 independent reflections
2136 reflections with *I* > 2σ(*I*)
*R*_int_ = 0.032
3 standard reflections every 200 reflections  intensity decay: 1%

#### Refinement

*R*[*F* ^2^ > 2σ(*F* ^2^)] = 0.054
*wR*(*F* ^2^) = 0.162
*S* = 1.01
3014 reflections
208 parameters
H-atom parameters constrained
Δρ_max_ = 0.26 e Å^−3^
Δρ_min_ = −0.32 e Å^−3^

### 

Data collection: *CAD-4 Software* (Enraf–Nonius, 1985 ▶); cell refinement: *CAD-4 Software*; data reduction: *XCAD4* (Harms & Wocadlo, 1995 ▶); program(s) used to solve structure: *SHELXS97* (Sheldrick, 2008 ▶); program(s) used to refine structure: *SHELXL97* (Sheldrick, 2008 ▶); molecular graphics: *SHELXTL* (Sheldrick, 2008 ▶); software used to prepare material for publication: *SHELXTL*.

## Supplementary Material

Crystal structure: contains datablocks I, global. DOI: 10.1107/S1600536811008440/vm2080sup1.cif

Structure factors: contains datablocks I. DOI: 10.1107/S1600536811008440/vm2080Isup2.hkl

Additional supplementary materials:  crystallographic information; 3D view; checkCIF report

## Figures and Tables

**Table 1:** Hydrogen-bond geometry (Å, °) *Cg*2 is the centroid of the C7–C12 ring.

*D*—H⋯*A*	*D*—H	H⋯*A*	*D*⋯*A*	*D*—H⋯*A*
O5—H5*A*⋯O6^i^	0.82	1.71	2.482 (3)	155
C14—H14*B*⋯*Cg*2^ii^	0.96	2.90	3.571 (4)	128

Symmetry codes: (i) 
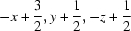
; (ii) 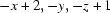.

**Fig. 1.**
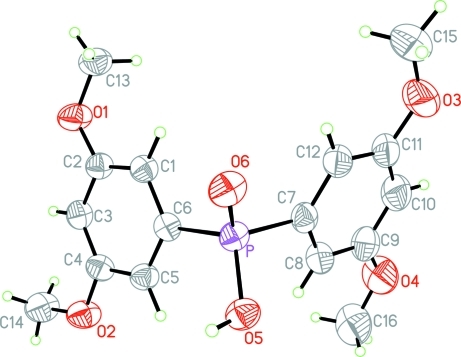
The molecular structure of (I) with displacement ellipsoids drawn at the 30% probability level.

**Fig. 2.**
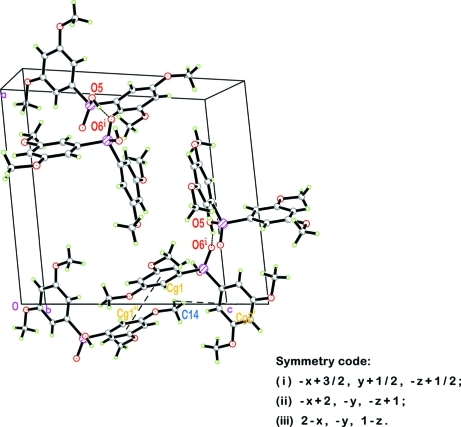
The crystal structure of (I). Dashed lines indicate hydrogen bonds or π—π interactions .

## supplementary crystallographic information

### Comment

The title compound, bis(3,5-dimethoxyphenyl)phosphinic acid (I) is an important intermediate for preparing metal phosphine complexes.

The molecular structure of (I) is shown in Fig. 1. The bond lengths and angles are within normal ranges (Allen *et al.*, 1987).

The dihedral angle between ring 1 (C1—C6) and ring 2 (C7—C12) is 73.5 (1)°. The P atom is situated close to the best planes through the benzene rings (deviation P of -0.054 (1) and 0.014 (1) Å for ring 1 and 2, respectively).

The C and O atoms of all methoxy groups lie very close to the mean planes of their attached rings. The C13 and C14 atoms of methoxy groups are 0.064 (1) and 0.1038 (1) Å, respectively, out of the C1–C6 mean plane. The C15 and C16 atoms are 0.055 (2) and 0.096 (1) Å, respectively, out of the C7–C8 mean plane.

In the crystal structure, intermolecular O—H···O interactions link the molecules into chains parallel to the b-direction (Table 1, Fig. 2). These chains are linked by C—H···π (Table 1) and π—π interactions [distance *Cg*1···*Cg*1^ii^ = 3.7307 (29) Å where *Cg*1 is the centroid of C1—C6; symmetry code ii: 2 - *x*,-*y*,1 - *z*] to give a three-dimensional network, which seems to be very effective in the stabilization of the crystal structure (Fig. 2).

### Experimental

The title compound was synthesized by the reaction of dimethoxyphenyl bromide, t-BuLi, *N*,*N*-dimethylphosphoramic dichloride in aqueous HCl and THF (Watson *et al.*, 2006). Crystals suitable for X-ray analysis were obtained by dissolving the title compound (50 mg) in ethyl acetate (10 ml) and evaporating the solvent slowly at room temperature for about 3 d.

### Refinement

H atoms were positioned geometrically, with O—H = 0.82 Å and C—H = 0.93 and 0.96 Å for aromatic and methoxy H, respectively, and constrained to ride on their parent atoms, with *U*_iso_(H) = *xU*_eq_(C), where *x* = 1.2 for phenyl H and *x* = 1.5 for all other H atoms.

### Crystal data

**Table:**

C_16_H_19_O_6_P	*F*(000) = 712
*M**_r_* = 338.28	*D*_x_ = 1.367 Mg m^−^^3^
Monoclinic, *P*2_1_/*n*	Mo *K*α radiation, λ = 0.71073 Å
Hall symbol: -P 2yn	Cell parameters from 25 reflections
*a* = 14.554 (3) Å	θ = 10–14°
*b* = 7.7620 (16) Å	µ = 0.20 mm^−^^1^
*c* = 14.634 (3) Å	*T* = 298 K
β = 96.14 (3)°	Block, colorless
*V* = 1643.7 (6) Å^3^	0.30 × 0.20 × 0.10 mm
*Z* = 4	

### Data collection

**Table:**

Enraf–Nonius CAD-4 diffractometer	2136 reflections with *I* > 2σ(*I*)
Radiation source: fine-focus sealed tube	*R*_int_ = 0.032
graphite	θ_max_ = 25.4°, θ_min_ = 1.9°
ω/2θ scans	*h* = 0→17
Absorption correction: ψ scan (North *et al.*, 1968)	*k* = 0→9
*T*_min_ = 0.944, *T*_max_ = 0.981	*l* = −17→17
3138 measured reflections	3 standard reflections every 200 reflections
3014 independent reflections	intensity decay: 1%

### Refinement

**Table:**

Refinement on *F*^2^	Primary atom site location: structure-invariant direct methods
Least-squares matrix: full	Secondary atom site location: difference Fourier map
*R*[*F*^2^ > 2σ(*F*^2^)] = 0.054	Hydrogen site location: inferred from neighbouring sites
*wR*(*F*^2^) = 0.162	H-atom parameters constrained
*S* = 1.01	*w* = 1/[σ^2^(*F*_o_^2^) + (0.095*P*)^2^] where *P* = (*F*_o_^2^ + 2*F*_c_^2^)/3
3014 reflections	(Δ/σ)_max_ < 0.001
208 parameters	Δρ_max_ = 0.26 e Å^−^^3^
0 restraints	Δρ_min_ = −0.31 e Å^−^^3^

### Special details

**Table:**

Geometry. All e.s.d.'s (except the e.s.d. in the dihedral angle between two l.s. planes) are estimated using the full covariance matrix. The cell e.s.d.'s are taken into account individually in the estimation of e.s.d.'s in distances, angles and torsion angles; correlations between e.s.d.'s in cell parameters are only used when they are defined by crystal symmetry. An approximate (isotropic) treatment of cell e.s.d.'s is used for estimating e.s.d.'s involving l.s. planes.
Refinement. Refinement of *F*^2^ against ALL reflections. The weighted *R*-factor *wR* and goodness of fit *S* are based on *F*^2^, conventional *R*-factors *R* are based on *F*, with *F* set to zero for negative *F*^2^. The threshold expression of *F*^2^ > σ(*F*^2^) is used only for calculating *R*-factors(gt) *etc*. and is not relevant to the choice of reflections for refinement. *R*-factors based on *F*^2^ are statistically about twice as large as those based on *F*, and *R*- factors based on ALL data will be even larger.

### Fractional atomic coordinates and isotropic or equivalent isotropic displacement parameters (Å^2^)

**Table:**

	*x*	*y*	*z*	*U*_iso_*/*U*_eq_	
P	0.84250 (5)	0.16050 (10)	0.25720 (5)	0.0347 (2)	
O1	0.83620 (18)	−0.2020 (3)	0.55369 (14)	0.0568 (7)	
C1	0.84208 (19)	−0.0319 (4)	0.41535 (19)	0.0369 (7)	
H1A	0.8151	−0.1182	0.3774	0.044*	
O2	0.96481 (18)	0.3582 (3)	0.57873 (15)	0.0582 (7)	
C2	0.8584 (2)	−0.0570 (4)	0.5091 (2)	0.0388 (7)	
O3	0.9727 (2)	−0.3021 (4)	0.0512 (2)	0.0853 (10)	
C3	0.8993 (2)	0.0712 (4)	0.5657 (2)	0.0417 (7)	
H3A	0.9099	0.0529	0.6287	0.050*	
O4	1.17640 (19)	0.1274 (4)	0.1644 (2)	0.0833 (9)	
C4	0.9241 (2)	0.2244 (4)	0.5293 (2)	0.0403 (7)	
O5	0.85561 (14)	0.3545 (3)	0.23979 (14)	0.0429 (5)	
H5A	0.8125	0.4084	0.2577	0.064*	
C5	0.9083 (2)	0.2521 (4)	0.4345 (2)	0.0397 (7)	
H5B	0.9256	0.3556	0.4093	0.048*	
O6	0.74981 (14)	0.0893 (3)	0.22249 (13)	0.0432 (5)	
C6	0.86672 (19)	0.1244 (4)	0.37875 (19)	0.0342 (7)	
C7	0.9317 (2)	0.0648 (4)	0.19870 (19)	0.0388 (7)	
C8	1.0195 (2)	0.1405 (4)	0.2072 (2)	0.0482 (8)	
H8A	1.0313	0.2393	0.2424	0.058*	
C9	1.0885 (2)	0.0655 (5)	0.1622 (2)	0.0570 (9)	
C10	1.0691 (3)	−0.0832 (5)	0.1110 (3)	0.0669 (11)	
H10A	1.1156	−0.1346	0.0816	0.080*	
C11	0.9820 (3)	−0.1564 (5)	0.1027 (2)	0.0577 (9)	
C12	0.9129 (2)	−0.0827 (4)	0.1475 (2)	0.0469 (8)	
H12A	0.8544	−0.1320	0.1431	0.056*	
C13	0.7992 (3)	−0.3423 (4)	0.5002 (2)	0.0583 (9)	
H13A	0.7871	−0.4362	0.5399	0.087*	
H13B	0.8426	−0.3782	0.4591	0.087*	
H13C	0.7426	−0.3076	0.4653	0.087*	
C14	0.9762 (3)	0.3417 (5)	0.6759 (2)	0.0683 (11)	
H14A	1.0051	0.4436	0.7026	0.102*	
H14B	1.0143	0.2434	0.6929	0.102*	
H14C	0.9169	0.3269	0.6979	0.102*	
C15	0.8843 (4)	−0.3867 (6)	0.0433 (3)	0.0889 (15)	
H15A	0.8867	−0.4879	0.0059	0.133*	
H15B	0.8379	−0.3097	0.0154	0.133*	
H15C	0.8695	−0.4190	0.1033	0.133*	
C16	1.1963 (3)	0.2843 (7)	0.2119 (4)	0.0937 (16)	
H16A	1.2597	0.3150	0.2083	0.141*	
H16B	1.1859	0.2707	0.2752	0.141*	
H16C	1.1569	0.3736	0.1845	0.141*	

### Atomic displacement parameters (Å^2^)

**Table:**

	*U*^11^	*U*^22^	*U*^33^	*U*^12^	*U*^13^	*U*^23^
P	0.0339 (4)	0.0366 (4)	0.0325 (4)	0.0010 (3)	−0.0014 (3)	0.0043 (3)
O1	0.0837 (18)	0.0470 (14)	0.0378 (12)	−0.0164 (12)	−0.0022 (11)	0.0083 (10)
C1	0.0355 (15)	0.0396 (16)	0.0347 (15)	−0.0025 (13)	−0.0010 (12)	−0.0011 (13)
O2	0.0813 (17)	0.0495 (14)	0.0405 (12)	−0.0168 (13)	−0.0085 (11)	−0.0039 (11)
C2	0.0410 (17)	0.0379 (17)	0.0370 (16)	−0.0002 (14)	0.0009 (13)	0.0034 (13)
O3	0.106 (2)	0.0688 (19)	0.088 (2)	−0.0052 (17)	0.0441 (18)	−0.0322 (16)
C3	0.0444 (17)	0.0488 (19)	0.0308 (15)	0.0022 (15)	−0.0016 (13)	0.0010 (14)
O4	0.0547 (16)	0.099 (2)	0.102 (2)	−0.0111 (16)	0.0322 (15)	−0.0187 (19)
C4	0.0409 (17)	0.0399 (17)	0.0384 (16)	−0.0004 (14)	−0.0039 (13)	−0.0040 (14)
O5	0.0405 (12)	0.0402 (12)	0.0480 (12)	0.0013 (10)	0.0044 (9)	0.0060 (10)
C5	0.0435 (17)	0.0362 (17)	0.0386 (16)	−0.0003 (14)	0.0014 (13)	0.0027 (14)
O6	0.0406 (12)	0.0478 (13)	0.0391 (11)	−0.0042 (10)	−0.0060 (9)	0.0079 (10)
C6	0.0290 (15)	0.0383 (16)	0.0345 (15)	0.0030 (12)	−0.0002 (12)	−0.0010 (13)
C7	0.0448 (18)	0.0412 (17)	0.0302 (15)	0.0056 (14)	0.0025 (13)	0.0041 (13)
C8	0.0483 (19)	0.054 (2)	0.0426 (17)	−0.0002 (16)	0.0067 (15)	−0.0035 (15)
C9	0.049 (2)	0.070 (2)	0.055 (2)	0.0012 (18)	0.0166 (16)	0.0011 (19)
C10	0.071 (3)	0.069 (3)	0.066 (2)	0.009 (2)	0.031 (2)	−0.004 (2)
C11	0.079 (3)	0.053 (2)	0.0446 (19)	0.003 (2)	0.0201 (18)	−0.0024 (17)
C12	0.052 (2)	0.0481 (19)	0.0411 (17)	0.0011 (16)	0.0081 (15)	0.0012 (16)
C13	0.077 (3)	0.047 (2)	0.051 (2)	−0.0154 (19)	0.0073 (18)	0.0025 (17)
C14	0.096 (3)	0.066 (3)	0.0411 (19)	−0.021 (2)	−0.0045 (19)	−0.0060 (19)
C15	0.118 (4)	0.065 (3)	0.086 (3)	−0.017 (3)	0.024 (3)	−0.025 (2)
C16	0.062 (3)	0.121 (4)	0.103 (4)	−0.027 (3)	0.030 (3)	−0.020 (3)

### Geometric parameters (Å, °)

**Table:**

P—O6	1.496 (2)	C7—C12	1.379 (4)
P—O5	1.542 (2)	C7—C8	1.400 (4)
P—C7	1.791 (3)	C8—C9	1.387 (5)
P—C6	1.798 (3)	C8—H8A	0.9300
O1—C2	1.357 (4)	C9—C10	1.389 (5)
O1—C13	1.414 (4)	C10—C11	1.383 (5)
C1—C2	1.381 (4)	C10—H10A	0.9300
C1—C6	1.389 (4)	C11—C12	1.381 (5)
C1—H1A	0.9300	C12—H12A	0.9300
O2—C4	1.365 (4)	C13—H13A	0.9600
O2—C14	1.419 (4)	C13—H13B	0.9600
C2—C3	1.388 (4)	C13—H13C	0.9600
O3—C11	1.358 (4)	C14—H14A	0.9600
O3—C15	1.437 (5)	C14—H14B	0.9600
C3—C4	1.367 (4)	C14—H14C	0.9600
C3—H3A	0.9300	C15—H15A	0.9600
O4—C9	1.363 (4)	C15—H15B	0.9600
O4—C16	1.417 (5)	C15—H15C	0.9600
C4—C5	1.397 (4)	C16—H16A	0.9600
O5—H5A	0.8200	C16—H16B	0.9600
C5—C6	1.382 (4)	C16—H16C	0.9600
C5—H5B	0.9300		
			
O6—P—O5	115.32 (12)	O4—C9—C10	116.3 (3)
O6—P—C7	110.96 (14)	C8—C9—C10	119.2 (3)
O5—P—C7	102.55 (13)	C11—C10—C9	121.4 (3)
O6—P—C6	110.65 (13)	C11—C10—H10A	119.3
O5—P—C6	107.51 (13)	C9—C10—H10A	119.3
C7—P—C6	109.45 (13)	O3—C11—C12	125.0 (4)
C2—O1—C13	117.9 (2)	O3—C11—C10	115.3 (3)
C2—C1—C6	118.9 (3)	C12—C11—C10	119.7 (3)
C2—C1—H1A	120.6	C7—C12—C11	119.3 (3)
C6—C1—H1A	120.6	C7—C12—H12A	120.3
C4—O2—C14	117.4 (3)	C11—C12—H12A	120.3
O1—C2—C1	124.8 (3)	O1—C13—H13A	109.5
O1—C2—C3	114.6 (3)	O1—C13—H13B	109.5
C1—C2—C3	120.6 (3)	H13A—C13—H13B	109.5
C11—O3—C15	117.4 (3)	O1—C13—H13C	109.5
C4—C3—C2	120.4 (3)	H13A—C13—H13C	109.5
C4—C3—H3A	119.8	H13B—C13—H13C	109.5
C2—C3—H3A	119.8	O2—C14—H14A	109.5
C9—O4—C16	117.2 (3)	O2—C14—H14B	109.5
O2—C4—C3	124.9 (3)	H14A—C14—H14B	109.5
O2—C4—C5	115.2 (3)	O2—C14—H14C	109.5
C3—C4—C5	119.9 (3)	H14A—C14—H14C	109.5
P—O5—H5A	109.5	H14B—C14—H14C	109.5
C6—C5—C4	119.4 (3)	O3—C15—H15A	109.5
C6—C5—H5B	120.3	O3—C15—H15B	109.5
C4—C5—H5B	120.3	H15A—C15—H15B	109.5
C5—C6—C1	120.9 (3)	O3—C15—H15C	109.5
C5—C6—P	120.0 (2)	H15A—C15—H15C	109.5
C1—C6—P	119.1 (2)	H15B—C15—H15C	109.5
C12—C7—C8	121.5 (3)	O4—C16—H16A	109.5
C12—C7—P	119.5 (2)	O4—C16—H16B	109.5
C8—C7—P	119.0 (2)	H16A—C16—H16B	109.5
C9—C8—C7	118.9 (3)	O4—C16—H16C	109.5
C9—C8—H8A	120.6	H16A—C16—H16C	109.5
C7—C8—H8A	120.6	H16B—C16—H16C	109.5
O4—C9—C8	124.5 (4)		
			
C13—O1—C2—C1	4.3 (5)	O6—P—C7—C12	−14.6 (3)
C13—O1—C2—C3	−176.4 (3)	O5—P—C7—C12	−138.3 (2)
C6—C1—C2—O1	178.8 (3)	C6—P—C7—C12	107.8 (3)
C6—C1—C2—C3	−0.5 (4)	O6—P—C7—C8	166.3 (2)
O1—C2—C3—C4	−179.4 (3)	O5—P—C7—C8	42.6 (3)
C1—C2—C3—C4	0.0 (5)	C6—P—C7—C8	−71.3 (3)
C14—O2—C4—C3	−4.7 (5)	C12—C7—C8—C9	0.6 (5)
C14—O2—C4—C5	175.5 (3)	P—C7—C8—C9	179.7 (2)
C2—C3—C4—O2	−179.9 (3)	C16—O4—C9—C8	−3.7 (6)
C2—C3—C4—C5	−0.1 (5)	C16—O4—C9—C10	176.5 (4)
O2—C4—C5—C6	−179.5 (3)	C7—C8—C9—O4	179.5 (3)
C3—C4—C5—C6	0.7 (5)	C7—C8—C9—C10	−0.7 (5)
C4—C5—C6—C1	−1.2 (4)	O4—C9—C10—C11	−179.1 (4)
C4—C5—C6—P	178.0 (2)	C8—C9—C10—C11	1.0 (6)
C2—C1—C6—C5	1.1 (4)	C15—O3—C11—C12	−0.6 (6)
C2—C1—C6—P	−178.1 (2)	C15—O3—C11—C10	177.8 (4)
O6—P—C6—C5	−139.0 (2)	C9—C10—C11—O3	−179.6 (3)
O5—P—C6—C5	−12.2 (3)	C9—C10—C11—C12	−1.2 (6)
C7—P—C6—C5	98.4 (3)	C8—C7—C12—C11	−0.7 (5)
O6—P—C6—C1	40.2 (3)	P—C7—C12—C11	−179.8 (2)
O5—P—C6—C1	167.0 (2)	O3—C11—C12—C7	179.2 (3)
C7—P—C6—C1	−82.3 (2)	C10—C11—C12—C7	1.0 (5)

### Hydrogen-bond geometry (Å, °)

**Table:**

Cg2 is the centroid of the C7–C12 ring.

**Table:**

*D*—H···*A*	*D*—H	H···*A*	*D*···*A*	*D*—H···*A*
O5—H5A···O6^i^	0.82	1.71	2.482 (3)	155
C14—H14B···Cg2^ii^	0.96	2.90	3.571 (4)	128

Symmetry codes: (i) −*x*+3/2, *y*+1/2, −*z*+1/2; (ii) −*x*+2, −*y*, −*z*+1.
